# Association between blood eosinophil count and 28-day mortality among critically ill patients with atrial fibrillation: A retrospective cohort study

**DOI:** 10.1097/MD.0000000000049796

**Published:** 2026-07-17

**Authors:** Zongjun Hu, Xiangjun Kong, Jianguo Chen, Shengyao Wang, Zhongjun Kang, Xia Huang

**Affiliations:** a Department of Critical Care Medicine, Chongqing University Three Gorges Hospital, Chongqing, People’s Republic of China; b Department of Cardiology, The Second People’s Hospital of Neijiang, Affiliated Neijiang Hospital of Southwest Medical University, Neijiang, Sichuan, People’s Republic of China; c Department of Cardiology, Ziyang Central Hospital, Ziyang hospital of West China Hospital, Sichuan University, Ziyang, Sichuan, People’s Republic of China.

**Keywords:** atrial fibrillation, blood eosinophil count, MIMIC-IV database, mortality

## Abstract

Blood eosinophil count has been associated with prognosis in patients with cardiovascular disease. However, its prognostic value among critically ill patients with atrial fibrillation (AF) remains unclear. This study aimed to investigate the association between admission blood eosinophil count and 28-day all-cause mortality in this population. This retrospective cohort study used data from the Medical Information Mart for Intensive Care IV database, version 3.1. Multivariable Cox regression, restricted cubic spline analysis, threshold-effect analysis, and Kaplan–Meier survival analysis were used to evaluate the association between blood eosinophil count and 28-day mortality. A total of 2787 critically ill patients with AF were included, of whom 1948 (69.9%) were male. The mean age was 70.4 ± 10.9 years, and the overall 28-day mortality rate was 9.8% (n = 273). Compared with patients in the lowest eosinophil count quartile (*Q*1), the adjusted hazard ratios for 28-day mortality were 0.51 (95% confidence interval [CI]: 0.37–0.70; *P* < .001) for *Q*2, 0.37 (95% CI: 0.25–0.55; *P* < .001) for *Q*3, and 0.45 (95% CI: 0.32–0.63; *P* < .001) for *Q*4. Restricted cubic spline analysis showed an L-shaped association between blood eosinophil count and 28-day mortality (*P* for nonlinearity <.001). Threshold-effect analysis identified an inflection point at 0.08 × 10^9^/L. Below this threshold, a higher eosinophil count was associated with lower 28-day mortality (hazard ratio per 0.01 × 10^9^/L increase, 0.864; 95% CI: 0.794–0.940; *P* < .001), whereas no significant association was observed above the threshold. In critically ill patients with AF, blood eosinophil count showed an L-shaped association with 28-day mortality. Lower eosinophil count was associated with a higher risk of short-term mortality.

## 1. Introduction

Atrial fibrillation (AF) is the most common sustained cardiac arrhythmia,^[1,2]^ with approximately 33.5 million cases worldwide in 2020^[3]^ and a prevalence projected to increase from 1.7% in 2020 to 2.4% by 2050.^[4]^ AF increases mortality, heart failure, stroke, and chronic kidney disease risk.^[5]^ In the intensive care unit (ICU), patients with AF have higher in-hospital mortality and longer stays than those without AF,^[6,7]^ challenging clinical management. Therefore, early risk assessment in critically ill patients with AF is important for stratification and decision-making.

Inflammation is involved in cardiovascular disease and may provide prognostic information in critically ill patients.^[8,9]^ Besides conventional inflammatory markers, cell-specific immunologic indicators may offer complementary information for risk stratification. Among these, eosinophils are recognized as immune cells involved in cardiovascular inflammation, repair, and thrombosis.^[10]^ Animal studies suggest eosinophils participate in myocardial repair and remodeling through inflammatory modulation and reparative signaling.^[11,12]^ Clinical studies show that low blood eosinophil counts at admission are associated with increased short-term mortality after myocardial infarction^[13,14]^ and in critically ill patients with heart failure.^[15]^ Although leukocyte differentials and eosinophils have been examined in relation to atrial fibrillation,^[16,17]^ the prognostic value of admission blood eosinophil count in critically ill patients with AF remains unclear. This gap motivated the present study.

This study evaluated whether admission blood eosinophil count is independently associated with 28-day mortality among critically ill patients with AF. We hypothesized that lower admission blood eosinophil count would be associated with higher short-term mortality.

## 2. Methods

### 2.1. Data source

This retrospective cohort study used data from the Medical Information Mart for Intensive Care IV (MIMIC-IV) database, version 3.1, which contains clinical data from 94,458 ICU stays between 2008 and 2022 at Beth Israel Deaconess Medical Center in Boston, Massachusetts.^[18]^ The creation of the MIMIC-IV database and its data-sharing initiative were approved by the Institutional Review Board of Beth Israel Deaconess Medical Center, with a waiver of informed consent. Because this study used de-identified data from a database available to credentialed users, additional ethical approval was not required. One investigator, Zongjun Hu, completed the required training and obtained access to the database with certification ID 60604021. This cohort study was conducted in accordance with the Strengthening the Reporting of Observational Studies in Epidemiology guidelines.

### 2.2. Study population

**Figure 1.**
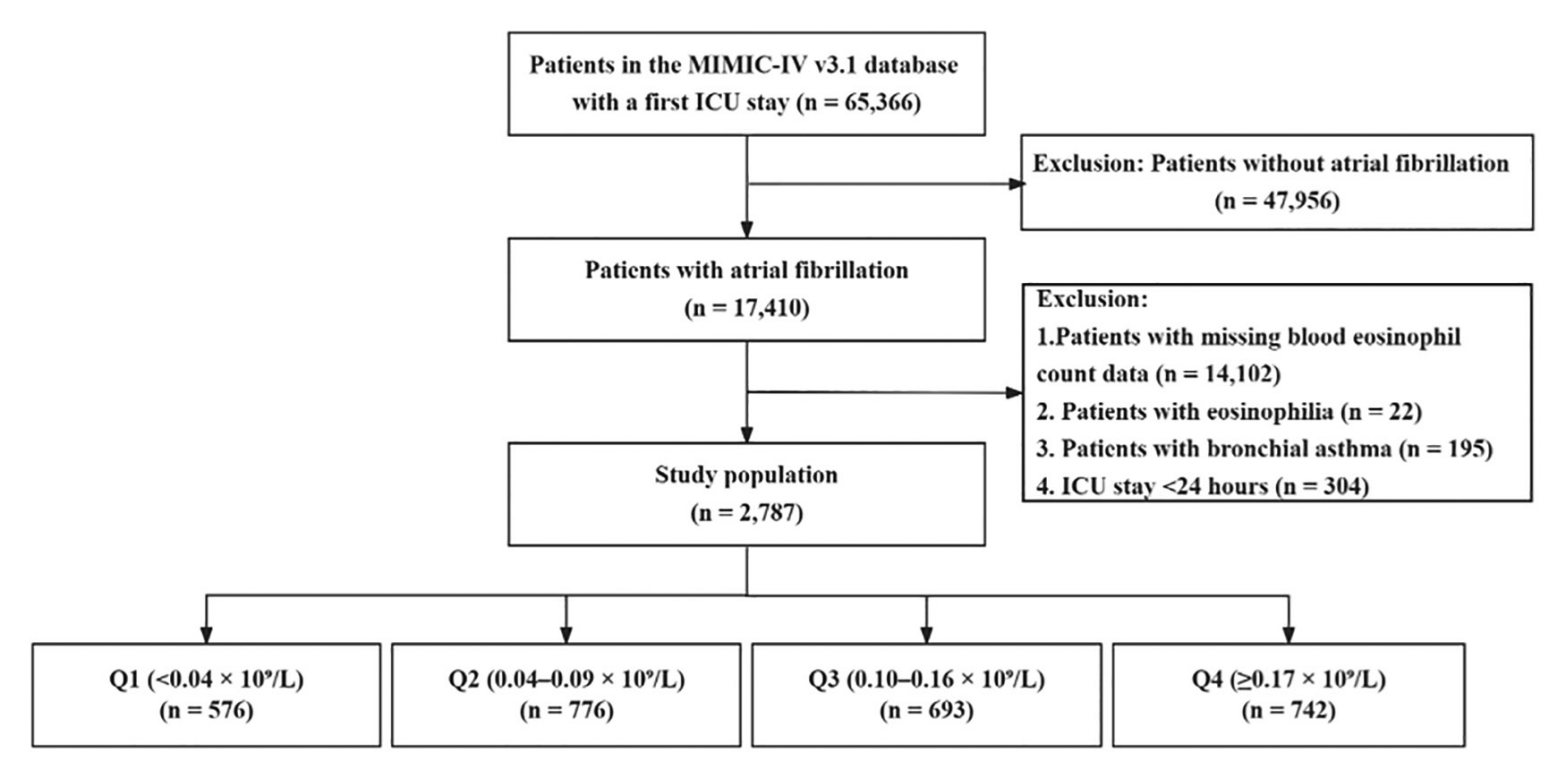
Flowchart of the study population. Eligible patients were further categorized into 4 groups according to admission blood eosinophil count quartiles. ICU = intensive care unit, MIMIC-IV = Medical Information Mart for Intensive Care IV.

Patients with AF admitted to the ICU were identified using the International Classification of Diseases, 9th Revision (ICD-9), and 10th Revision (ICD-10) codes.^[19]^ For patients with multiple ICU stays, only data from the 1st ICU stay were included. Patients were excluded if they had missing blood eosinophil count data, an ICU stay shorter than 24 hours, or a documented diagnosis of eosinophilia or bronchial asthma (Fig. 1).

### 2.3. Data extraction and outcome

Navicat Premium 17.0 (PremiumSoft CyberTech Ltd., Hong Kong, China) was used to extract data using Structured Query Language, with Structured Query Language code adapted from the MIMIC code repository. Baseline characteristics within the 1st 24 hours after ICU admission were collected, including demographic characteristics such as sex and age. Disease severity was assessed using the Sequential Organ Failure Assessment, Charlson Comorbidity Index (CCI), Acute Physiology Score III, and Simplified Acute Physiology Score II (SAPS II).

Vital signs included systolic blood pressure, diastolic blood pressure (DBP), heart rate, respiratory rate, and peripheral oxygen saturation. Laboratory variables included white blood cell count, blood eosinophil count, hemoglobin, platelet count, creatinine, blood urea nitrogen, bicarbonate, sodium, chloride, and potassium. Therapeutic interventions during hospitalization were also recorded, including mechanical ventilation, renal replacement therapy (RRT), angiotensin-converting enzyme inhibitors, angiotensin receptor blockers, inotropic agents, diuretics, and β-blockers. Detailed classifications of these medications are provided in Table S1, Supplemental Digital Content 1. Comorbidities were ascertained using documented ICD-9 and ICD-10 codes and included chronic kidney disease, malignant cancer, hypertension, diabetes mellitus, anemia, cerebrovascular disease, severe liver disease, congestive heart failure, and myocardial infarction. In this cohort, the proportion of missing data for all examined variables was <1% (Table S2, Supplemental Digital Content 2); therefore, no imputation was performed.

The study endpoint was 28-day all-cause mortality, a standard endpoint in critical care research that captures the acute phase of ICU illness and is widely used in studies based on the MIMIC-IV database. Date-of-death information was obtained from hospital records and state death records linked to the MIMIC-IV database.

### 2.4. Statistical analysis

Critically ill patients with AF were categorized into 4 groups according to admission blood eosinophil count quartiles. Baseline demographic and clinical characteristics were summarized for each group. Continuous variables are presented as mean ± standard deviation or median (interquartile range), as appropriate, and categorical variables are presented as frequencies and percentages. Continuous variables were compared using 1-way analysis of variance or the Kruskal–Wallis test, as appropriate. Categorical variables were compared using Pearson chi-square test when the expected cell counts were adequate; otherwise, Fisher exact test was used.

Multivariable Cox proportional hazards regression models were used to evaluate the association between admission blood eosinophil count and 28-day mortality in critically ill patients with AF. Results are presented as hazard ratios (HRs) with 95% confidence intervals (CIs). Covariates were selected for adjustment if they changed the estimated HR by ≥10% or were considered clinically relevant based on previous literature and clinical practice. Four models were constructed: the crude model was unadjusted; model 1 was adjusted for age and sex; model 2 was further adjusted for myocardial infarction, congestive heart failure, cerebrovascular disease, malignant cancer, severe liver disease, chronic kidney disease, diabetes mellitus, anemia, CCI, and SAPS II; and model 3 was further adjusted for heart rate, DBP, white blood cell count, hemoglobin, platelet count, creatinine, blood urea nitrogen, bicarbonate, sodium, mechanical ventilation, RRT, angiotensin-converting enzyme inhibitor/angiotensin receptor blocker use, inotropic drug use, diuretic use, and β-blocker use.

Kaplan–Meier survival curves were generated according to admission blood eosinophil count quartiles, and differences were compared using the log-rank test. The potential nonlinear association between admission blood eosinophil count and 28-day mortality was assessed using restricted cubic spline (RCS) analysis with 4 knots placed at the 5th, 35th, 65th, and 95th percentiles, with blood eosinophil count treated as a continuous variable. The reference point for the RCS curve was set at the cohort median of 0.10 × 10^9^/L (HR = 1.0). Following RCS analysis, a 2-piecewise Cox proportional hazards regression model was used to identify potential threshold effects and estimate the inflection point. In the threshold-effect analysis, HRs were calculated per 0.01 × 10^9^/L increase in blood eosinophil count. This threshold-effect analysis was adjusted for all covariates included in model 3.

Effect modification was assessed through stratified analyses according to sex, age dichotomized at 65 years, and key comorbidities, including myocardial infarction, chronic kidney disease, congestive heart failure, and diabetes mellitus. Interaction tests were used to assess whether the association between admission blood eosinophil count and 28-day mortality differed across subgroups. All analyses were conducted using R software, version 4.2.2 (The R Foundation for Statistical Computing, Vienna, Austria), and the Free Statistics analysis platform, version 2.1.1 (Beijing Free Clinical Medical Technology Co., Ltd.). A 2-sided *P* value <.05 was considered statistically significant.

## 3. Results

### 3.1. Baseline characteristics of the study population

This study included 2787 critically ill patients with AF, with a mean age of 70.4 ± 10.9 years; 1948 patients (69.9%) were male. Patients were divided into 4 groups according to admission blood eosinophil count: *Q*1 (<0.04 × 10^9^/L), *Q*2 (0.04–0.09 × 10^9^/L), *Q*3 (0.10–0.16 × 10^9^/L), and *Q*4 (≥0.17 × 10^9^/L). Baseline characteristics according to blood eosinophil count quartiles are summarized in Table 1.

**Table 1:** Baseline characteristics of patients according to admission blood eosinophil count quartiles.

Categories	Blood eosinophil count (×10^9^/L)					*P* value
	Total	*Q*1 (<0.04)	*Q*2 (0.04–0.09)	*Q*3 (0.10–0.16)	*Q*4 (≥0.17)	
	N = 2787	N = 576	N = 776	N = 693	N = 742	
Demographics						
Male, n (%)	1948 (69.9)	354 (61.5)	548 (70.6)	512 (73.9)	534 (72.0)	<.001
Age (yr)	70.4 ± 10.9	71.2 ± 12.6	70.3 ± 10.6	70.5 ± 9.9	69.8 ± 10.5	.116
Comorbidities, n (%)						
Myocardial infarction	769 (27.6)	156 (27.1)	203 (26.2)	190 (27.4)	220 (29.6)	.484
Congestive heart failure	1150 (41.3)	308 (53.5)	289 (37.2)	241 (34.8)	312 (42.0)	<.001
Cerebrovascular disease	417 (15.0)	109 (18.9)	124 (16.0)	91 (13.1)	93 (12.5)	.005
Malignant cancer	175 (6.3)	59 (10.2)	39 (5.0)	25 (3.6)	52 (7.0)	<.001
Severe liver disease	57 (2.0)	18 (3.1)	13 (1.7)	8 (1.2)	18 (2.4)	.067
Chronic kidney disease	732 (26.3)	159 (27.6)	183 (23.6)	175 (25.3)	215 (29.0)	.086
Diabetes mellitus	985 (35.3)	182 (31.6)	266 (34.3)	240 (34.6)	297 (40.0)	.011
Anemia	1986 (71.3)	365 (63.4)	571 (73.6)	505 (72.9)	545 (73.5)	<.001
Hypertension	1191 (42.7)	199 (34.5)	361 (46.5)	310 (44.7)	321 (43.3)	<.001
Severity scores						
Charlson Comorbidity Index	6.0 (4.0, 8.0)	6.0 (5.0, 8.0)	6.0 (4.0, 7.0)	6.0 (4.0, 7.0)	6.0 (4.0, 8.0)	<.001
APS III	39.0 (29.0, 55.0)	48.0 (34.8, 67.0)	38.0 (29.0, 51.0)	36.0 (27.0, 50.0)	38.0 (29.0, 53.0)	<.001
SAPS II	38.0 (32.0, 46.0)	40.0 (34.0, 50.0)	37.0 (31.0, 44.0)	37.0 (31.0, 45.0)	38.0 (32.0, 46.0)	<.001
SOFA	5.0 (3.5, 8.0)	6.0 (4.0, 9.0)	5.0 (3.0, 7.0)	5.0 (4.0, 8.0)	5.0 (4.0, 8.0)	.001
Vital signs						
Heart rate (beats/min)	82.3 ± 13.8	85.8 ± 18.1	81.5 ± 12.8	80.6 ± 10.9	81.8 ± 12.9	<.001
SBP (mm Hg)	113.1 ± 12.2	113.7 ± 14.8	113.4 ± 12.0	112.8 ± 10.9	112.4 ± 11.3	.203
DBP (mm Hg)	60.1 ± 9.7	62.7 ± 10.6	60.4 ± 10.0	58.8 ± 8.8	59.0 ± 9.1	<.001
Respiratory rate (breaths/min)	18.8 ± 3.1	19.6 ± 3.5	18.7 ± 3.0	18.3 ± 2.8	18.6 ± 3.1	<.001
SpO_2_ (%)	97.2 ± 1.8	97.0 ± 2.1	97.3 ± 1.7	97.3 ± 1.8	97.2 ± 1.7	.003
Laboratory tests						
White blood cells (10^9^/L)	11.8 (8.8, 15.5)	11.0 (8.1, 15.4)	10.8 (8.1, 14.4)	11.8 (9.1, 14.7)	13.5 (10.1, 17.7)	<.001
Hemoglobin (g/dL)	9.8 ± 2.1	10.3 ± 2.4	9.7 ± 2.1	9.5 ± 1.8	9.6 ± 1.9	<.001
Platelet count (10^9^/L)	144.0 (111.0, 191.0)	157.0 (110.0, 223.5)	136.5 (105.0, 181.8)	134.0 (107.0, 171.0)	152.0 (119.0, 192.8)	<.001
Creatinine (mg/dL)	0.9 (0.8, 1.3)	1.0 (0.8, 1.5)	0.9 (0.7, 1.2)	0.9 (0.7, 1.2)	1.0 (0.8, 1.3)	<.001
Urea nitrogen (mg/dL)	17.0 (13.0, 25.0)	21.0 (14.0, 36.0)	16.0 (13.0, 23.0)	16.0 (13.0, 22.0)	18.0 (14.0, 25.0)	<.001
Bicarbonate (mmol/L)	22.4 ± 3.4	22.0 ± 4.5	22.3 ± 3.1	22.4 ± 2.9	22.6 ± 3.3	.003
Sodium (mmol/L)	138.0 ± 4.1	137.8 ± 5.6	137.9 ± 3.8	138.3 ± 3.5	138.1 ± 3.5	.224
Chloride (mmol/L)	105.7 ± 5.8	103.4 ± 7.2	106.1 ± 5.6	106.8 ± 5.1	105.9 ± 5.0	<.001
Potassium (mmol/L)	4.4 ± 0.6	4.4 ± 0.8	4.4 ± 0.6	4.4 ± 0.6	4.4 ± 0.6	.730
Treatments, n (%)						
Mechanical ventilation	1730 (62.1)	328 (56.9)	457 (58.9)	463 (66.8)	482 (65.0)	<.001
RRT	202 (7.2)	53 (9.2)	54 (7.0)	33 (4.8)	62 (8.4)	.011
ACEI/ARB use	357 (12.8)	73 (12.7)	117 (15.1)	81 (11.7)	86 (11.6)	.148
β-blocker use	2056 (73.8)	387 (67.2)	589 (75.9)	531 (76.6)	549 (74.0)	<.001
Diuretic use	2166 (77.7)	389 (67.5)	612 (78.9)	572 (82.5)	593 (79.9)	<.001
Inotropic drug use	1220 (43.8)	308 (53.5)	306 (39.4)	268 (38.7)	338 (45.6)	<.001

Data are presented as mean ± standard deviation, median (interquartile range), or n (%), as appropriate. *P* values were calculated using 1-way analysis of variance (ANOVA) or the Kruskal–Wallis test for continuous variables and Pearson chi-square test or Fisher exact test for categorical variables.

ACEI = angiotensin-converting enzyme inhibitor, APS III = Acute Physiology Score III, ARB = angiotensin receptor blocker, DBP = diastolic blood pressure, RRT = renal replacement therapy, SAPS II = Simplified Acute Physiology Score II, SBP = systolic blood pressure, SOFA = Sequential Organ Failure Assessment, SpO_2_ = peripheral oxygen saturation.

Age did not differ significantly across the 4 groups. Compared with patients in the higher blood eosinophil count quartiles, those in *Q*1 had a lower proportion of males and a higher prevalence of congestive heart failure, cerebrovascular disease, and malignant cancer. Patients in *Q*1 also had a higher heart rate, DBP, respiratory rate, Acute Physiology Score III, SAPS II, Sequential Organ Failure Assessment score, and CCI, indicating more severe illness and a greater comorbidity burden among patients with lower blood eosinophil counts.

The 28-day mortality rate differed significantly across blood eosinophil count quartiles (*P* < .001). Patients in *Q*1 had the highest 28-day mortality rate (21.9%), compared with *Q*2 (7.5%), *Q*3 (4.5%), and *Q*4 (7.8%) (Table S3, Supplemental Digital Content 3).

### 3.2. Association between blood eosinophil count and 28-day mortality

Multivariable Cox proportional hazards regression analysis showed that higher blood eosinophil count quartiles were associated with lower 28-day mortality compared with the lowest blood eosinophil count quartile (Table 2). In the fully adjusted model (model 3), the HRs for 28-day mortality were 0.51 (95% CI: 0.37–0.70; *P* < .001) for *Q*2, 0.37 (95% CI: 0.25–0.55; *P* < .001) for *Q*3, and 0.45 (95% CI: 0.32–0.63; *P* < .001) for *Q*4. The trend across blood eosinophil count quartiles was statistically significant (*P* for trend <.001).

**Table 2:** Association between admission blood eosinophil count quartiles and 28-day mortality in critically ill patients with atrial fibrillation.

Variables	Crude model		Model 1		Model 2		Model 3	
	HR (95% CI)	*P* value	HR (95% CI)	*P* value	HR (95% CI)	*P* value	HR (95% CI)	*P* value
Blood eosinophil count (×10^9^/L)								
*Q*1	1.00 (Ref)		1.00 (Ref)		1.00 (Ref)		1.00 (Ref)	
*Q*2	0.31 (0.23–0.43)	<.001	0.34 (0.25–0.46)	<.001	0.48 (0.35–0.66)	<.001	0.51 (0.37–0.70)	<.001
*Q*3	0.18 (0.12–0.27)	<.001	0.20 (0.14–0.30)	<.001	0.30 (0.20–0.45)	<.001	0.37 (0.25–0.55)	<.001
*Q*4	0.33 (0.24–0.45)	<.001	0.36 (0.26–0.49)	<.001	0.45 (0.33–0.62)	<.001	0.45 (0.32–0.63)	<.001
*P* for trend		<.001		<.001		<.001		<.001

Cox proportional hazards regression models were used to calculate hazard ratios (HRs) with 95% confidence intervals (CIs). The crude model was unadjusted. Model 1 was adjusted for age and sex. Model 2 was adjusted for covariates in Model 1 plus myocardial infarction, congestive heart failure, cerebrovascular disease, malignant cancer, severe liver disease, chronic kidney disease, diabetes mellitus, anemia, Charlson Comorbidity Index, and SAPS II. Model 3 was adjusted for covariates in Model 2 plus heart rate, DBP, white blood cell count, hemoglobin, platelet count, creatinine, blood urea nitrogen, bicarbonate, sodium, mechanical ventilation, RRT, ACEI/ARB use, β-blocker use, diuretic use, and inotropic drug use.

ACEI = angiotensin-converting enzyme inhibitor, ARB = angiotensin receptor blocker, CI = confidence interval, DBP = diastolic blood pressure, HR = hazard ratio, RRT = renal replacement therapy, SAPS II = Simplified Acute Physiology Score II, SBP = systolic blood pressure.

**Figure 2.**
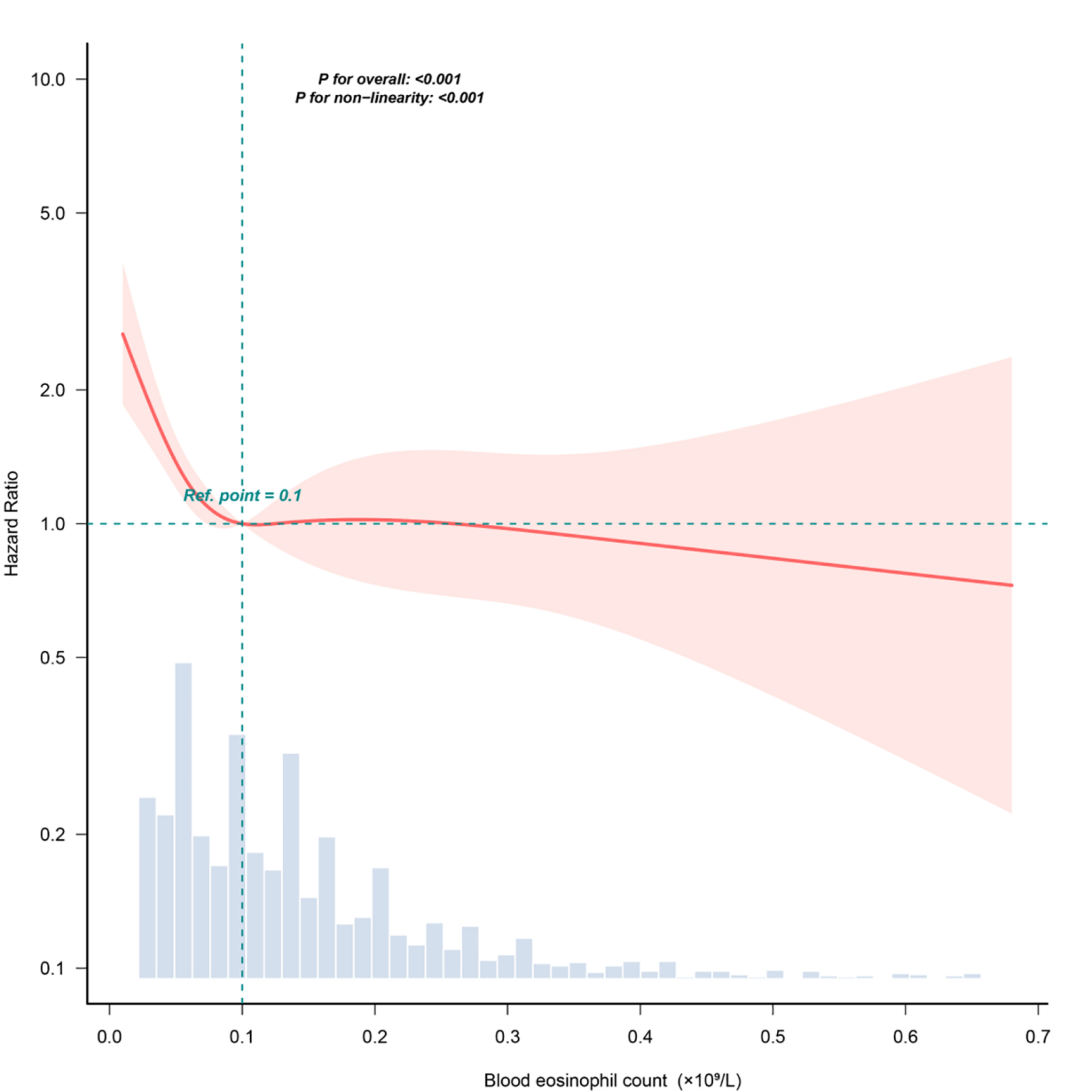
Nonlinear association between admission blood eosinophil count and 28-day mortality assessed by restricted cubic spline analysis. The reference point was set at 0.10 × 10^9^/L. The model was adjusted for age, sex, myocardial infarction, congestive heart failure, cerebrovascular disease, malignant cancer, severe liver disease, chronic kidney disease, diabetes mellitus, anemia, Charlson Comorbidity Index, SAPS II, heart rate, DBP, white blood cell count, hemoglobin, platelet count, creatinine, blood urea nitrogen, bicarbonate, sodium, mechanical ventilation, RRT, ACEI/ARB use, β-blocker use, diuretic use, and inotropic drug use. The solid line represents the adjusted hazard ratio, and the shaded area represents the 95% confidence interval. Only the central 99% of observations are displayed. ACEI = angiotensin-converting enzyme inhibitor, ARB = angiotensin receptor blocker, DBP = diastolic blood pressure, SAPS II = Simplified Acute Physiology Score II.

RCS analysis showed an L-shaped association between blood eosinophil count and 28-day mortality (*P* for nonlinearity <.001; Fig. 2). Threshold-effect analysis identified an inflection point at 0.08 × 10^9^/L (likelihood ratio test, *P* < .001). Below this threshold, a higher blood eosinophil count was inversely associated with 28-day mortality (HR per 0.01 × 10^9^/L increase, 0.864; 95% CI: 0.794–0.940; *P* < .001). Above this threshold, the association was not statistically significant (HR per 0.01 × 10^9^/L increase, 0.989; 95% CI: 0.970–1.009; *P* = .281) (Table 3).

**Table 3:** Threshold-effect analysis of the association between blood eosinophil count and 28-day mortality.

Blood eosinophil count	HR	95% CI	*P* value
< 0.08 × 10^9^/L	0.864	(0.794–0.940)	<.001
≥ 0.08 × 10^9^/L	0.989	(0.970–1.009)	.281
Likelihood ratio test			<.001

Adjusted for all covariates in Model 3. HRs were calculated per 0.01 × 10^9^/L increase.

CI, confidence interval, HR, hazard ratio.

### 3.3. Kaplan–Meier survival curve analysis

**Figure 3.**
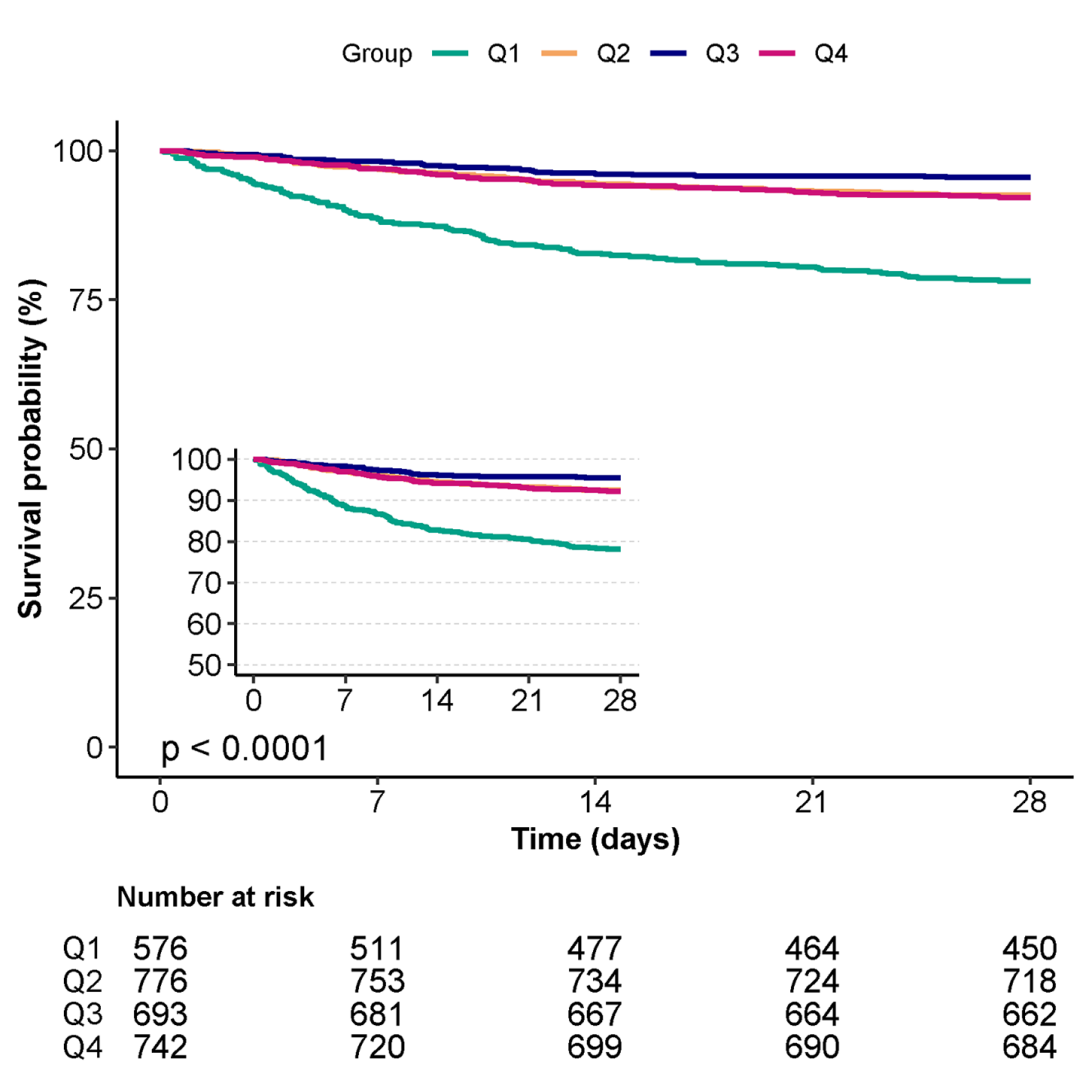
Kaplan–Meier curves for 28-day survival according to admission blood eosinophil count quartiles in critically ill patients with atrial fibrillation.

Kaplan–Meier survival curves according to blood eosinophil count quartiles are shown in Figure 3. Patients in the lowest blood eosinophil count quartile had significantly lower 28-day survival than those in the other quartiles (log-rank *P* < .001).

### 3.4. Subgroup analysis

**Figure 4.**
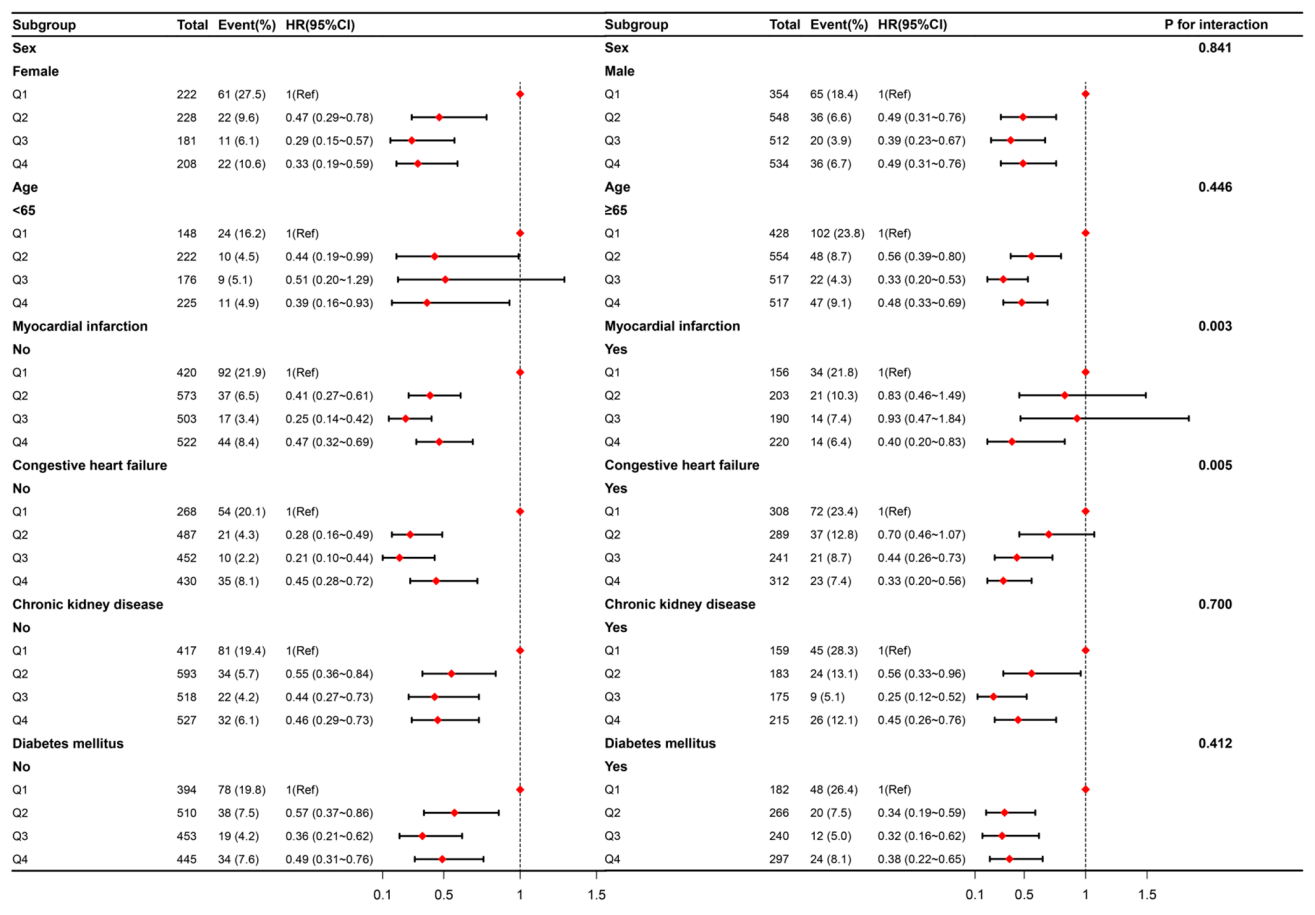
Subgroup analyses of the association between admission blood eosinophil count quartiles and 28-day mortality in critically ill patients with atrial fibrillation. *Q*1 was used as the reference group within each subgroup, and hazard ratios for *Q*2, *Q*3, and *Q*4 are presented. Each stratified model was adjusted for all covariates included in model 3, except for the corresponding stratification variable. *P* values for interaction were calculated to assess whether the association differed across subgroups. CI = confidence interval, HR = hazard ratio.

Subgroup analyses showed that the association between blood eosinophil count and 28-day mortality was generally consistent across strata defined by sex, age, chronic kidney disease, and diabetes mellitus (Fig. 4). However, significant interactions were observed for myocardial infarction and congestive heart failure. The *P* values for interaction were .003 for myocardial infarction and .005 for congestive heart failure, suggesting that the association between blood eosinophil count and 28-day mortality may differ according to the presence of these conditions.

## 4. Discussion

In this retrospective cohort study of critically ill patients with AF, admission blood eosinophil count was independently and inversely associated with 28-day all-cause mortality. This association persisted after adjusting for demographics, comorbidities, disease severity, vital signs, laboratory parameters, and treatments. Cox regression showed that higher eosinophil count quartiles had a lower 28-day mortality risk than the lowest quartile. RCS analysis identified an L-shaped association: below the inflection point, a higher eosinophil count was associated with lower mortality; above it, no significant association was observed. Subgroup analyses showed interactions for myocardial infarction and congestive heart failure, suggesting the association may vary by cardiovascular comorbidity.

Studies have reported associations between eosinophil count and cardiovascular disease, though the direction differs across settings. In chronic atherosclerotic or thrombotic conditions, a higher eosinophil count may be associated with adverse outcomes.^[20,21]^ In acute cardiovascular and critical illness settings, a lower eosinophil count has been associated with worse outcomes. Vural et al reported lower eosinophil counts at admission among ICU patients with acute decompensated heart failure who developed major adverse cardiovascular events,^[22]^ and a reduced eosinophil count after myocardial infarction has been associated with increased short-term mortality.^[13]^ Consistent with this pattern, the present study extends evidence to critically ill patients with AF, showing that eosinophil count at ICU admission was inversely associated with 28-day mortality.

These observations suggest that the prognostic implications of eosinophil count are context-dependent. In chronic conditions, eosinophil count may contribute to inflammatory or thrombotic risk, whereas in acute illness, eosinopenia may reflect impaired immune-regulatory or tissue-reparative capacity and greater vulnerability to physiological stress. Thus, the association between eosinophil count and mortality in critically ill patients with AF may partly reflect acute immune-inflammatory dysregulation beyond illness severity.

AF is an inflammation-related arrhythmia.^[23]^ Systemic inflammation may promote atrial structural and electrical remodeling through oxidative stress, inflammasome signaling, and extracellular matrix deposition, contributing to AF persistence and recurrence.^[24–26]^ In critically ill patients, acute inflammation and hemodynamic stress may further increase atrial substrate instability and worsen outcomes. Recent studies support the relevance of inflammatory and cardiac markers in AF recurrence and prognosis. Karataş et al reported that the uric acid-to-albumin ratio was associated with AF recurrence after cryoballoon catheter ablation.^[27]^ Kalenderoğlu et al found that the uric acid-to-albumin ratio, systemic immune-inflammation index, and C-reactive protein-to-albumin ratio were associated with AF recurrence after cryoablation.^[28]^ Hayiroğlu et al showed that AF occurrence after dual-chamber permanent pacemaker implantation was associated with long-term prognosis in octogenarians.^[29]^ These findings suggest that inflammatory activity is associated with AF recurrence and adverse outcomes, supporting the relevance of inflammatory biomarkers in AF.

In this context, eosinopenia may have several explanations. Acute illness involves neuroendocrine activation, with elevated glucocorticoids and catecholamines that may induce eosinophil redistribution from the circulation, tissue sequestration, or apoptosis.^[30,31]^ This stress eosinopenia reflects a physiological response to systemic stress rather than bone marrow failure, providing an endocrine-immune context for the observed inverse association between eosinophil count and mortality.

Beyond stress, low eosinophil count may also reflect reduced cardiac reparative capacity. Eosinophils support cardiac repair through type 2 immune pathways. Liu et al showed that eosinophil-derived interleukin-4 protects cardiomyocytes from oxidative stress-induced death,^[11]^ while Toor et al demonstrated that eosinophil deficiency impairs anti-inflammatory macrophage polarization and increases scar formation, reversible by interleukin-4 therapy.^[12]^ In AF patients, where atrial remodeling and fibrosis are central pathological features, reduced reparative signaling may impair atrial tissue homeostasis and increase vulnerability to adverse outcomes. Additionally, eosinophils may modulate hemostatic balance through interactions with platelets and thrombomodulin function,^[20,32]^ relevant in AF patients who already carry an elevated thromboembolic risk.

The L-shaped association indicates a nonlinear pattern: mortality risk increased markedly below the threshold, but no additional benefit was observed above it. Very low eosinophil counts may identify patients with pronounced stress eosinopenia and reduced immune-reparative capacity, while counts above the threshold may indicate relatively preserved homeostatic or reparative capacity.

Several limitations should be noted. First, this retrospective study cannot establish causality; unmeasured confounders may remain despite multivariable adjustment. Second, only admission eosinophil count was analyzed; subsequent changes during ICU stay were not assessed. Third, eosinophil count may be affected by medications and conditions such as glucocorticoids, immunosuppression, infection, allergy, or hematologic disorders. Though we adjusted for available variables, these factors could not be fully excluded. Fourth, causes of death were unavailable in the MIMIC-IV database, preventing cardiovascular versus non-cardiovascular distinction. Fifth, data from a single center limit generalizability. Finally, ICD-based AF identification may introduce misclassification.

## 5. Conclusion

In summary, admission blood eosinophil count showed an L-shaped association with 28-day mortality in critically ill patients with AF. Lower eosinophil counts were associated with higher short-term mortality risk. Eosinophil count, a readily available laboratory parameter, may help identify high-risk patients in this population. Prospective studies are needed to confirm these findings and clarify the underlying biological mechanisms.

## Acknowledgments

We thank the Laboratory for Computational Physiology at the Massachusetts Institute of Technology and Beth Israel Deaconess Medical Center for developing and maintaining the MIMIC-IV database and for making it available for research.

## Author contributions

**Conceptualization:** Zongjun Hu, Xiangjun Kong, Jianguo Chen.

**Data curation:** Zongjun Hu, Jianguo Chen, Shengyao Wang, Zhongjun Kang.

**Formal analysis:** Zongjun Hu, Shengyao Wang, Zhongjun Kang, Xia Huang.

**Methodology:** Zongjun Hu, Xiangjun Kong.

**Project administration:** Xiangjun Kong.

**Resources:** Xiangjun Kong.

**Software:** Zongjun Hu.

**Supervision:** Xiangjun Kong.

**Validation:** Xiangjun Kong.

**Visualization:** Zongjun Hu, Shengyao Wang, Zhongjun Kang.

**Writing – original draft:** Zongjun Hu, Jianguo Chen, Zhongjun Kang, Xia Huang.

**Writing – review & editing:** Xiangjun Kong, Shengyao Wang, Zhongjun Kang, Xia Huang.






