# Supplementary material for: Association between blood eosinophil count and 28-day mortality among critically ill patients with atrial fibrillation: A retrospective cohort study
Source: Medicine (Baltimore). 2026 Jul 17;105(29):e49796. doi: 10.1097/MD.0000000000049796 (PMC13384553; doi:10.1097/MD.0000000000049796)
Supplement: Supplementary file 1 [file medi-105-e49796-s001.docx]

**Table S1**. List of medications administered during ICU admission

| **ACEI** | | |  |
| --- | --- | --- | --- |
| Fosinopril | Captopril | Lisinopril | Ramipril |
| Enalapril |  |  |  |
| **ARB** |  |  |  |
| Olmesartan | Irbesartan | Losartan Potassium | Valsartan |
| **β-blocker** |  |  |  |
| Atenolol | Bisoprolol | Carvedilol | Labetalol |
| Metoprolol | Propranolol |  |  |
| **Diuretics** | |  |  |
| Mannitol 20% | Acetazolamide | Furosemide | Hydrochlorothiazide |
| Spironolactone |  |  |  |
| **Inotropic drugs** | | |  |
| Digoxin | Dopamine | Milrinone | Epinephrine |
| Dobutamine |  |  |  |
