# Supplementary material for: Association between blood eosinophil count and 28-day mortality among critically ill patients with atrial fibrillation: A retrospective cohort study
Source: Medicine (Baltimore). 2026 Jul 17;105(29):e49796. doi: 10.1097/MD.0000000000049796 (PMC13384553; doi:10.1097/MD.0000000000049796)
Supplement: Supplementary file 2 [file medi-105-e49796-s002.docx]

**Table S2**. Percentage of missing data for each variable

| **Variables** | **Missing count** | **Missing rate (%)** |
| --- | --- | --- |
| SBP | 13 | 0.46 |
| DBP | 13 | 0.46 |
| White blood cells | 4 | 0.14 |
| Hemoglobin | 3 | 0.11 |
| Platelet count | 12 | 0.43 |
| Creatinine | 3 | 0.11 |
| Urea nitrogen | 2 | 0.07 |
| Bicarbonate | 3 | 0.11 |
| Sodium | 6 | 0.21 |
| Chloride | 2 | 0.07 |
| Potassium | 11 | 0.39 |

SBP, systolic blood pressure; DBP, diastolic blood pressure.
