# Supplementary material for: Association between blood eosinophil count and 28-day mortality among critically ill patients with atrial fibrillation: A retrospective cohort study
Source: Medicine (Baltimore). 2026 Jul 17;105(29):e49796. doi: 10.1097/MD.0000000000049796 (PMC13384553; doi:10.1097/MD.0000000000049796)
Supplement: Supplementary file 3 [file medi-105-e49796-s003.docx]

**Table S3**. 28-day mortality across eosinophil quartiles

|  | Total (n= 2787) | Q1 (n= 576) | Q2 (n= 776) | Q3 (n= 693) | Q4 (n= 742) | *P* value |
| --- | --- | --- | --- | --- | --- | --- |
| 28-day mortality | 273 (9.8) | 126 (21.9) | 58 (7.5) | 31 (4.5) | 58 (7.8) | < 0.001 |

Eosinophil counts are expressed in ×10⁹/L. Q1, <0.04; Q2, 0.04–0.09; Q3, 0.10–0.16; Q4, ≥0.17. The *P* value was calculated using Pearson’s chi-square test.
